# Eye, Ear, Nose and Throat

**Published:** 1903-02

**Authors:** 

**Affiliations:** Brownwood, Texas


### Eye, Ear, Nose and Throat.

EDITED BY DR. P. M. PAYNE, BROWNWOOD, TEXAS.

Every good physician in Texas should read the splendid article of Dr. A. Duane, of New York, entitled "Some Considerations on the Hygienic and Prophylactic Treatment of Myopia." He says, "patient should employ the full correction of his myopia all the time both for distance and near work. Proper attention should be paid to illumination. In low and medium myopia moderate restriction of near work with momentary rest of the eyes at intervals should be advised, while in high and progressive myopia more rigid restriction of near work with plenty of out of door exercise should be insisted upon." He also strongly advises that patient be reexamined at frequent intervals to determine the progress, if any, of the myopia.—Reprinted from *New York Medical Journal* of June 7, 1902.

---

Dr. Lee Wallace Dean, in *American Medicine* of January 17th, writes interestingly on the "Therapy of Otitis Media Suppurative Chronica." His article shows an extensive research into the literature of the subject. He quotes fourteen times from Politzer and frequently from Deuch, Randall, Beck of Berlin and many others. His recapitulation is good, but he might have added that each case is a law unto itself, though certain it is that much valuable time is often lost by temporizing with some of the seemingly simple cases.

---

Dr. T. E. Hopkins, in December *Laryngoscope,* reports a case of "Foreign Body in Right Bronchus." A toy called a squawker ("a wooden tube two inches long, somewhat tapering; greatest diameter 7-16 of an inch. To the smaller end is attached a bag of thin rubber, which is inflated by blowing through the tube")—in other words, the noisy balloon of the circus-man. Tracheotomy was done, and the foreign body found in right bronchus and removed with long tracheal forceps tube end up. Uneventful recovery.

---

"Recent Progress in Laryngology, Otology and Rhinology" is the title of Dr. G. Hudson Makuen's address as chairman of that section of the American Medical Association at Saratoga Springs, N. Y., June 10-13, 1902. Speaking of adenoids he says: "The drift of opinion is more and more toward the early and complete removal of the hypertrophied pharyngeal tonsil." He says that Dr. O. Stewart reports the removal of one adenoid mass measuring three-eighths of an inch in length by three-fourths of an inch in depth from a child eleven days old with immediate relief of the symptoms, which were snoring and inability to nurse." Under head of "Foreign Bodies," he said that Dr. Roaldes had reported the results of special study of the technique of removing metallic foreign bodies from the air-passages by means of a magnet. Dr. Roaldes thinks that improvement of the technic would make it possible to remove such foreign bodies from the bronchus through the mouth by means of the combined use of strong magnet and flouroscopy and bronchoscopy. That portion of his address which dealt with the mastoid contains the following remarks: "The indications for surgical intervention in mastoid disease are not yet well defined, for the symptoms bear no distinct relation to the pathologic conditions within the mastoid process." He says Dr. G. Alexander reports eleven cases of mastoid operation in Politzer's clinic under cocaine anesthesia, and so successful was the procedure that he recommends the method in all cases where the co-operation of the patient can be secured.—Reported in *Journal of the American Medical Association,* January 17, 1903.

